# Acid Dissolution of Magnesian-Calcite Stromatolites from Lagoa Salgada: An Experimental Evaluation Using X‑ray Microtomography

**DOI:** 10.1021/acsomega.5c11036

**Published:** 2026-03-26

**Authors:** Vitor Felipe Hage Serra, Jamilly Pina da Silva, Ana Carolina Silva da Cunha, Jair Rodrigues Neyra, Cláudio Regis dos Santos Lucas, Daniel Nobre Nunes da Silva, Pedro Tupã Pandava Aum

**Affiliations:** †Graduate Program of Geophysics (CPGF), ‡Geology and Geochemistry Graduation Program (PPGG), 37871 Federal University of Pará (UFPA), Belém 66075-110, Brazil; § Energy and Petroleum Science Laboratory (LCPETRO), Engineering College, Salinópolis Campus, Federal University of Pará (UFPA), Salinópolis 68721-000, Brazil

## Abstract

The dissolution behavior of carbonate rocks in acidic environments is a fundamental process in reactive flow through porous media, with key applications in reservoir stimulation and carbon storage. Although many studies have examined dissolution kinetics in homogeneous rocks dominated by calcite or dolomite, the influence of compositional and structural heterogeneity remains poorly constrained, particularly in microbial carbonates typical of Brazil’s Pre-Salt reservoirs. This study investigates the acid dissolution of a magnesian-calcite stromatolite, considered an analog of the Barra Velha Formation, and compares it with two reference materials: Indiana Limestone and Silurian Dolomite. Static dissolution experiments were performed using 0.5 M hydrochloric acid. Dissolution behavior was quantified through average reaction rates and complemented by high-resolution X-ray microtomography, which enabled visualization and quantification of internal morphological evolution during the experiments. Results reveal substantial variation among stromatolitic facies, with dissolution rates increasing from Layer A to Layer C despite an opposite trend in magnesian-calcite content. Layer C exhibited the most extensive structural alteration and greatest mass loss, whereas Layer A showed slower kinetics and minimal modification. Indiana Limestone displayed intermediate behavior, while Silurian Dolomite was the least reactive. These findings demonstrate that, under the experimental conditions investigated and considering the stromatolite samples and fluid system used, microstructure and pore architecture exert a stronger control on carbonate dissolution rates than mineralogical composition alone under conditions of negligible fluid movement.

## Graphical abstract

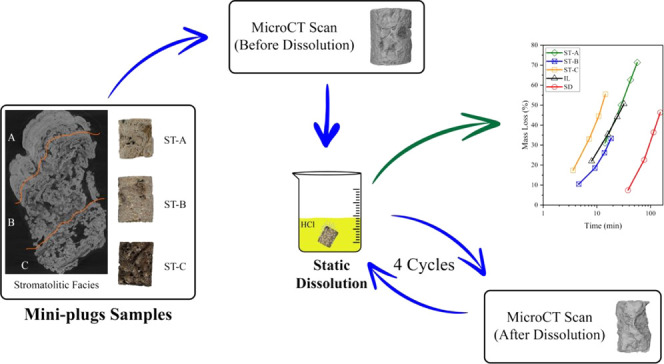

## 1 Introduction

The dissolution behavior of carbonate rocks in acidic environments is a fundamental process in numerous geological and engineering contexts, including reservoir diagenesis, carbon storage, and acid stimulation treatments in hydrocarbon production.^1–6^ The reaction kinetics governing carbonate dissolution are inherently complex, influenced not only by petrophysical parameters such as porosity and permeability but also by mineralogical composition and crystallochemical characteristics. In particular, the incorporation of foreign cations, such as magnesium, into the carbonate crystal lattice significantly alters the structural properties of these minerals, thereby modulating their reactivity under acid exposure.^7–14^

Understanding the factors that control carbonate reactivity is particularly important in heterogeneous systems, such as microbial carbonate reservoirs, where textural, compositional, and structural variations occur over small spatial scales.^15,16^ The Pre-Salt reservoirs of the South Atlantic margin, exemplified by the Barra Velha Formation in the Santos Basin, are dominated by complex microbial carbonates characterized by stromatolitic structures, vuggy porosity, and mineralogical heterogeneity.^17,18^ Due to strong heterogeneity, early stage well stimulation is often employed to enhance reservoir performance.^19^ Among the most widely adopted stimulation techniques is carbonate acidizing, which involves the injection of acidic solutions, typically hydrochloric acid, into the reservoir. The acid reacts with the carbonate matrix, dissolving part of the rock and increasing pore connectivity in the near-wellbore region. This enhanced connectivity improves well-reservoir communication and can significantly boost production rates.^20–22^

In this context, several studies have investigated the dissolution behavior of carbonate rocks in acidic solutions.^7,9,11–14,23–26^ Andriamihaja et al. (2016)^27^ investigated the dissolution kinetics of three carbonate microfacies: moldic muddy-wackestone, boundstone, and grainstone, under acidic conditions at 25 °C, 50 °C, and 75 °C, developing predictive models that highlighted the influence of porosity and crystal size on dissolution rates while finding permeability negligible. Their study revealed a two-phase dissolution behavior, with an initial rapid phase driven by reactive minerals followed by a slower, steady phase, emphasizing the role of microfacies-specific textures and temperature. Expanding on this, the authors^7^ examined static dissolution’s impact on 3D pore networks in argillaceous limestone and grain limestone, demonstrating that dissolution significantly enhances porosity and connectivity, particularly in grain limestone due to its higher calcite content. While pore throat modifications (radius and length) were minor, they led to drastic permeability improvements, underscoring rock heterogeneity and mineral composition as dominant controls over temperature.

Jora et al. (2021)^28^ studied six carbonate rocks (including limestones and dolomites) under static acid dissolution using HCl and acetic acid (HAc). Combining gravimetric analysis, NMR (T_2_ relaxation), and micro-CT imaging, they found that reactivity depended more on dolomitization (Mg content) than porosity/permeability. High-Mg rocks (e.g., Silurian dolomites, Edwards Brown) dissolved slower, forming a low-reactivity group, while low-Mg samples (e.g., Indiana Limestone, Desert Pink) showed high reactivity. HCl caused uniform surface erosion, whereas HAc preserved geometry but created roughness due to differing proton release mechanisms. NMR and micro-CT revealed minimal internal pore changes, indicating surface-dominated reactions under static conditions.

Wang et al. (2026) investigated CO_2_ ex-situ mineralization usingcrushed samples of the Lagoa Salgada stromatolite, separated into layers, in aqueous systems with sodium formate and reported a significant increase in mineral dissolution, especially due to the enhanced release of Ca^2+^ into solution.^29^

Although several studies have evaluated carbonate dissolution in acidic environments, most have focused on relatively homogeneous, monomineralic samples composed primarily of calcite or dolomite. While such materials are suitable for controlled laboratory experiments, they fail to represent the complexity of highly heterogeneous carbonate systems—particularly those associated with microbialites (stromatolite), as found in Brazil’s Pre-Salt reservoirs. To the best of our knowledge, no study has specifically examined the static acid dissolution behavior of Pre-Salt analogs like the stromatolites from Lagoa Salgada, RJ. In this work, we investigate the mineralogical and petrophysical controls on acid reactivity in stromatolitic carbonates through static hydrochloric acid dissolution experiments, using high-resolution X-ray microtomography to track morphological changes. Results are compared with those from Indiana Limestone and Silurian Dolomite standards to assess the influence of mineralogy, crystallographic structure, and textural heterogeneity on dissolution behavior in these complex carbonate systems.

## 2 Methodology

### 2.1 Carbonate Samples

This study examines three types of carbonate rocks: two homogeneous standards and one heterogeneous microbialite subdivided into three distinct facies. The standard samples, Indiana Limestone (IL) and Silurian Dolomite (SD) were obtained from Kocurek Industries Inc. (USA). Indiana Limestone is a Mississippian-aged limestone from the Salem Formation (Illinois Basin), composed of >98% calcite with interparticle porosity. SD originates from the Silurian-aged Lockport Dolomite (Michigan Basin), consisting of >98% dolomite with crystalline textures and higher permeability (typically 50–200 mD) due to its dolomitic fabric. The Lagoa Salgada stromatolitic samples (ST) were classified into three texturally and compositionally distinct facies: A, B, and C, according to previous studies.^15^ These layers exhibit complex mineralogy, including magnesian calcite, quartz, amorphous silica, and accessory phases such as hematite and rutile. Biogenic components like microfossils and serpulid tubes further contribute to the heterogeneity of their pore architecture. The pores architecture of the Lagoa Salgada stromatolite is heterogeneous, comprising regions with a fine-grained matrix characterized by smaller and less connected pores, as well as columnar zones with vuggy porosity, exhibiting larger and better-connected pore networks.^30^ Notably, these stromatolites are considered important analogs to the microbial carbonates of the Barra Velha Formation, Pre-Salt of the Santos Basin^31^ as supported by later petrophysical and geochemical studies.^15,32,33^ The geographic location and macroscopic features of the Lagoa Salgada stromatolite outcrops are illustrated in Figure 1.

**Figure 1:**
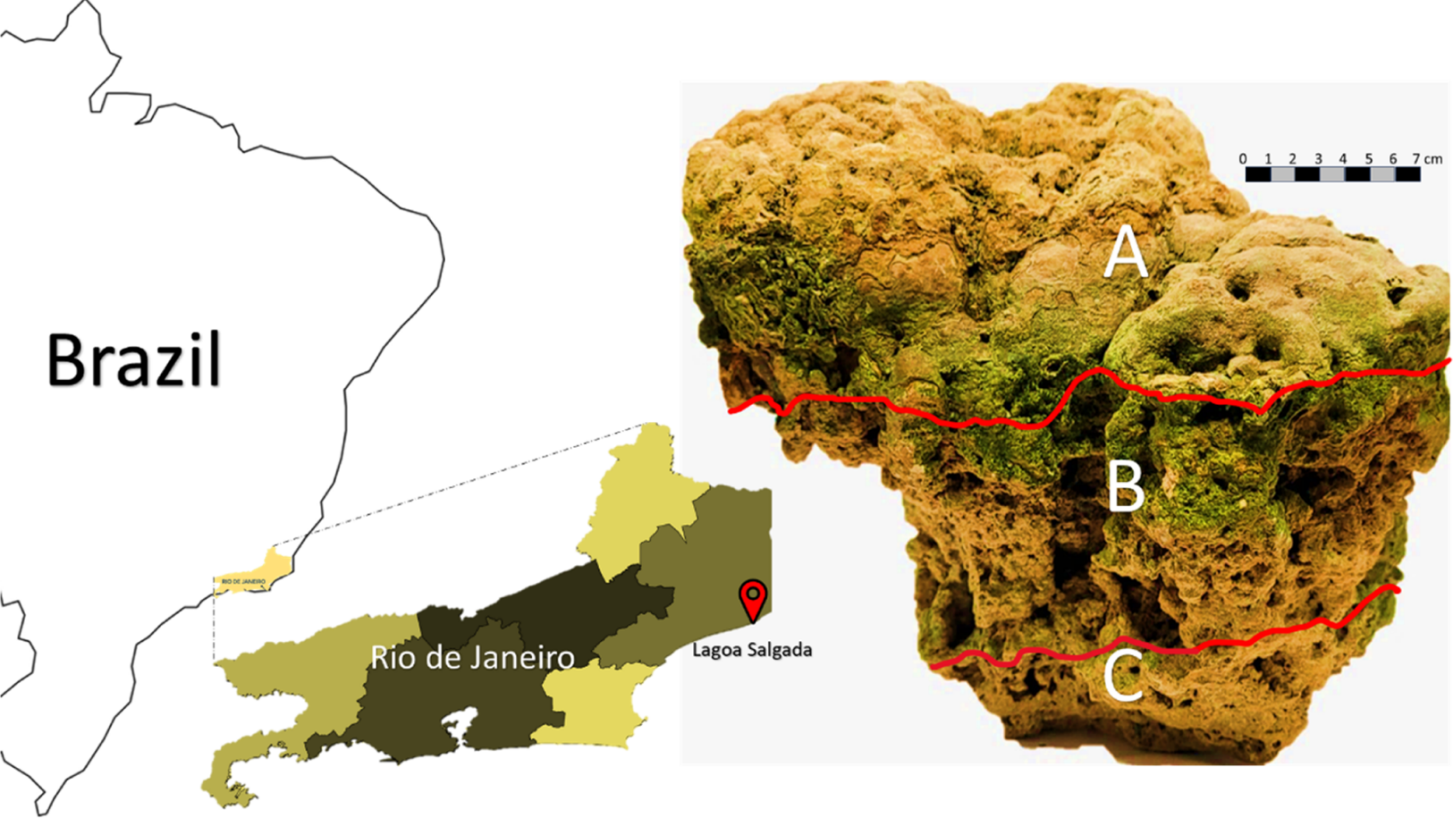
Location of the Lagoa Salgada stromatolitic carbonate. The photograph on the right shows the characteristic macroscopic zonation of the sampled material, subdivided into three distinct layers (A–C).

### 2.2 Compositional and Mineralogical Analyses

Representative aliquots from each stromatolitic layer were pulverized (<200 mesh) and previously characterized through X-ray diffraction (XRD) and X-ray fluorescence (XRF) analyses to determine bulk mineral composition. Detailed analytical procedures and mineralogical data sets for these materials were previously reported.^29^

In the present study, mineralogical information was used exclusively to quantify the relative abundance of the dominant carbonate phases, particularly magnesian calcite and dolomite, which were employed as compositional reference parameters for interpreting dissolution behavior. Minor and accessory phases were not considered in the quantitative analysis because they do not significantly contribute to the acid–rock reaction under the experimental conditions investigated.

### 2.3 Micro-CT Image Acquisition

Image acquisition was conducted using a General Electric (GE) Phoenix V|tome|x s 240 X-ray microtomograph, and image reconstruction was performed using GE’s datos|x software suite. Postprocessing and analysis were conducted in Thermo Fisher Scientific’s Avizo platform (version 2024.2), where Volume Rendering and Ortho Slice modules were used to enable three-dimensional visualization and two-dimensional slice comparisons. The pore space was segmented using a threshold-based segmentation method, allowing for the extraction of digital porosity data, which were quantified through the Label Analysis module.

The entire stromatolite sample (Figure 3a), used for the contextual and facies-scale analysis, was imaged using the following acquisition parameters: 140 kV voltage, 160 μA current, 200 ms exposure time, 2500 projections, and a spatial resolution of 113 μm.

For the high-resolution imaging of the acid dissolution experiments, cylindrical miniplugs (Indiana Limestone and Silurian Dolomite) and discrete facies samples from the stromatolite (Facies A, B, and C) were imaged before and after each acidification cycle. The acquisition parameters for these scans were conducted under the following ranges: 70–140 kV voltage, 70 to 160 μA current, 200 to 500 ms exposure time, 1100 to 1800 projections, and a spatial resolution ranging from 7 to 10 μm. All scans were performed in monoscan mode with 8 or 9 bhc corrections applied, depending on the data set.

### 2.4 Static Dissolution Experiments

The static dissolution experiments were conducted using cylindrical subsamples (mini-plugs) measuring 8 mm in diameter and 10 mm in length, extracted from the main rock samples, with the stromatolite (ST) plugs taken from visually distinct layer intervals. To ensure experimental consistency, samples underwent 24 h pretreatment with 35% hydrogen peroxide to remove organic residues, followed by distilled water rinsing and oven-drying at 200 °C for 5 h.

The dissolution experiments were carried out using a 0.5 mol/L HCl acid solution, prepared with ultrapure water (1.22 MΩ·cm, 0.81 μS/cm) and stored under ambient temperature conditions. Since the rock samples exhibit different reactivities, the *t*_50_ parameter, defined as the time required to reduce the sample mass to 50% of its original weight through dissolution,^28^ was initially estimated. This parameter was used to determine the time at which each dissolution cycle would be interrupted, ensuring a resolution consistent with the dissolution rate.

The experiments were conducted in triplicate, with each sample submerged in the acid solution until one-quarter of the *t*_50_ was reached. At the end of each dissolution cycle, the reaction was systematically halted to allow sample washing under running water, followed by drying. Simultaneously, the pH of the effluent from each cycle was measured using a portable pH meter (CDS107, Ômega). Monitoring these pH variations throughout the experiment provided a proxy for the mass loss of the rock matrix resulting solely from chemical dissolution. A flowchart outlining the major steps of the dissolution experiment is illustrated in Figure 2.

**Figure 2:**
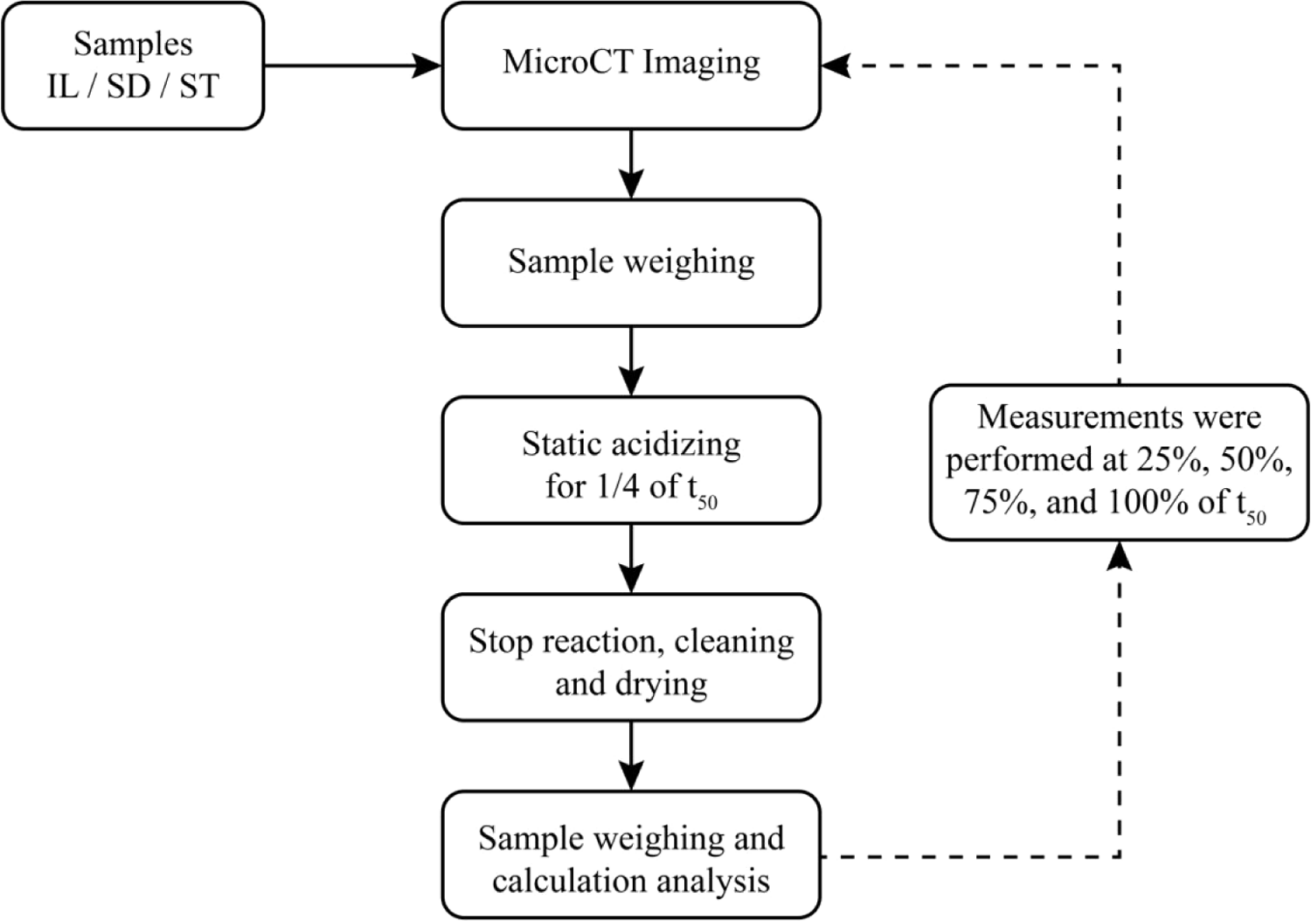
Experimental workflow for carbonate acid dissolution analysis assisted by Micro-CT imaging.

## 3 Results and Discussion

### 3.1 Porosity and Mineralogical Characterization

High-resolution X-ray microcomputed tomography (micro-CT) was employed to investigate the internal structural features and pore systems of the microbialite samples at a submillimetric scale and to visualize skeletal components. This approach enabled direct comparison among the different microbialite facies (A, B, and C). Transversal slices along the XZ plane (Figure 3) reveal facies-specific lamination styles and structural configurations. Facies A (Figure 3b) is characterized by visible micritic laminations occurring as laterally continuous bodies (biostromes) and coalescent or lenticular domical portions (bioherms), which are only slightly altered by natural dissolution in the lagoon environment. This facies also contains skeletal material and thin, discontinuous fenestral porosity that follows the orientation of the stromatolitic lamination (Figure 3b).

**Figure 3:**
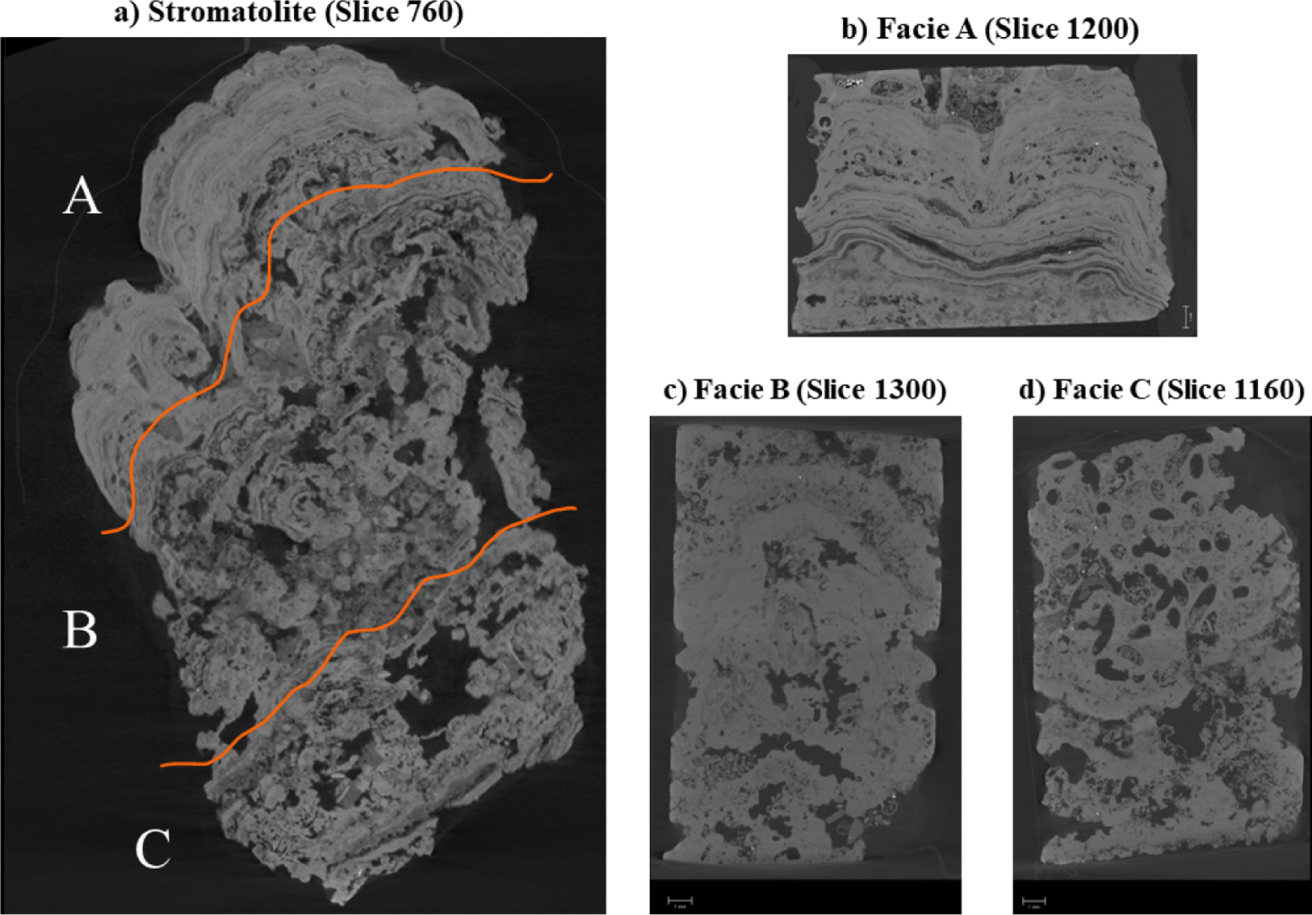
X-ray micro-CT slices (XZ plane) illustrating internal fabric variations among microbialite facies. (a) Whole-core stromatolite for contextual reference; (b) Facies A; (c) Facies B and (d) Facies C. All images share the same vertical orientation.

Facies B (Figure 3c), interpreted as a thrombolytic texture, it presents a massive framework with a lumpy appearance dominated by a vuggy porosity and rare preserved microbial laminations, in addition to a microfabric composed of hairy aggregates that exhibit the appearance of clots and lumps and a massive micritic matrix. The porous system of this facies is formed by a predominance of vug pores and pores produced by bioturbation, with local occurrences of interparticles, intraparticles, channel and moldic pores. The vug pores exhibit equidimensional irregular shapes ranging from 100 to 1000 μm and are distributed throughout the thrombolytic facies. Facies C (Figure 3d) is characterized by a vuggy stromatolitic framework, with pervasive macroporosity that obscures primary lamination and disrupts the original sedimentary fabric. This fabric destruction indicates significant post-depositional alteration and the development of enhanced fluid flow pathways.

Multiplanar sections (XY, XZ, YZ) of the samples are shown in Figures 4 and 6. In Facies A (Figure 4), the pore architecture is predominantly planar and occurs preferentially along grain laminations. The pore system is mainly composed of vug-type pores, followed by interparticle, intraparticle, and locally fenestral and moldic pores. The vugs are irregular in shape, ranging from approximately 200 to 600 μm, and are randomly distributed throughout the facies.

**Figure 4:**
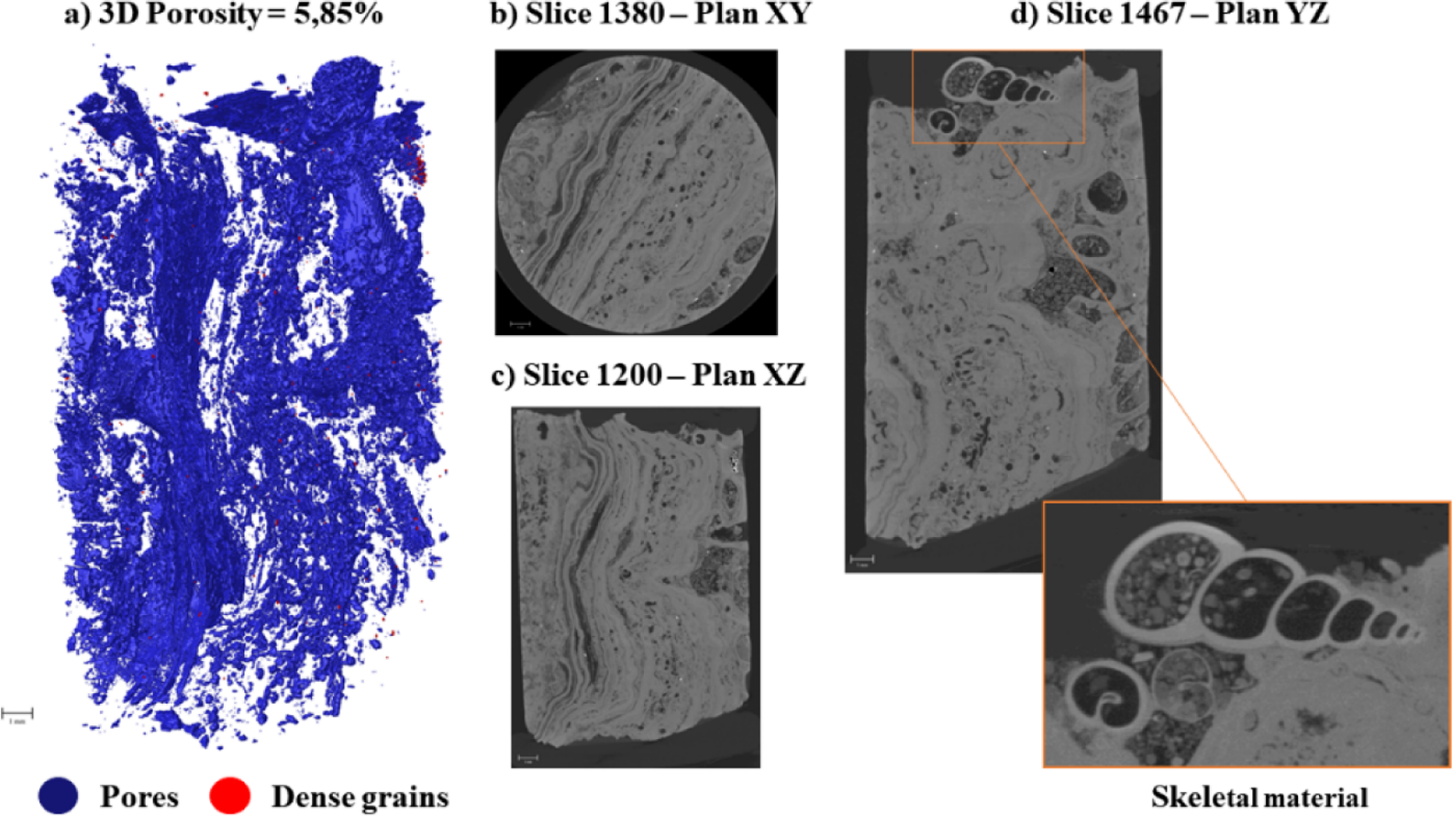
Pore architecture of Facies A resolved by micro-CT multiplanar views.

Facies B (Figure 5) displays a more heterogeneous pore system, including large and irregular cavities that contribute to a greater total pore volume relative to Facies A. Both facies exhibit abundant skeletal remains, primarily micritized bioclasts, which locally generate moldic porosity (Figures 4d and 5d).

**Figure 5:**
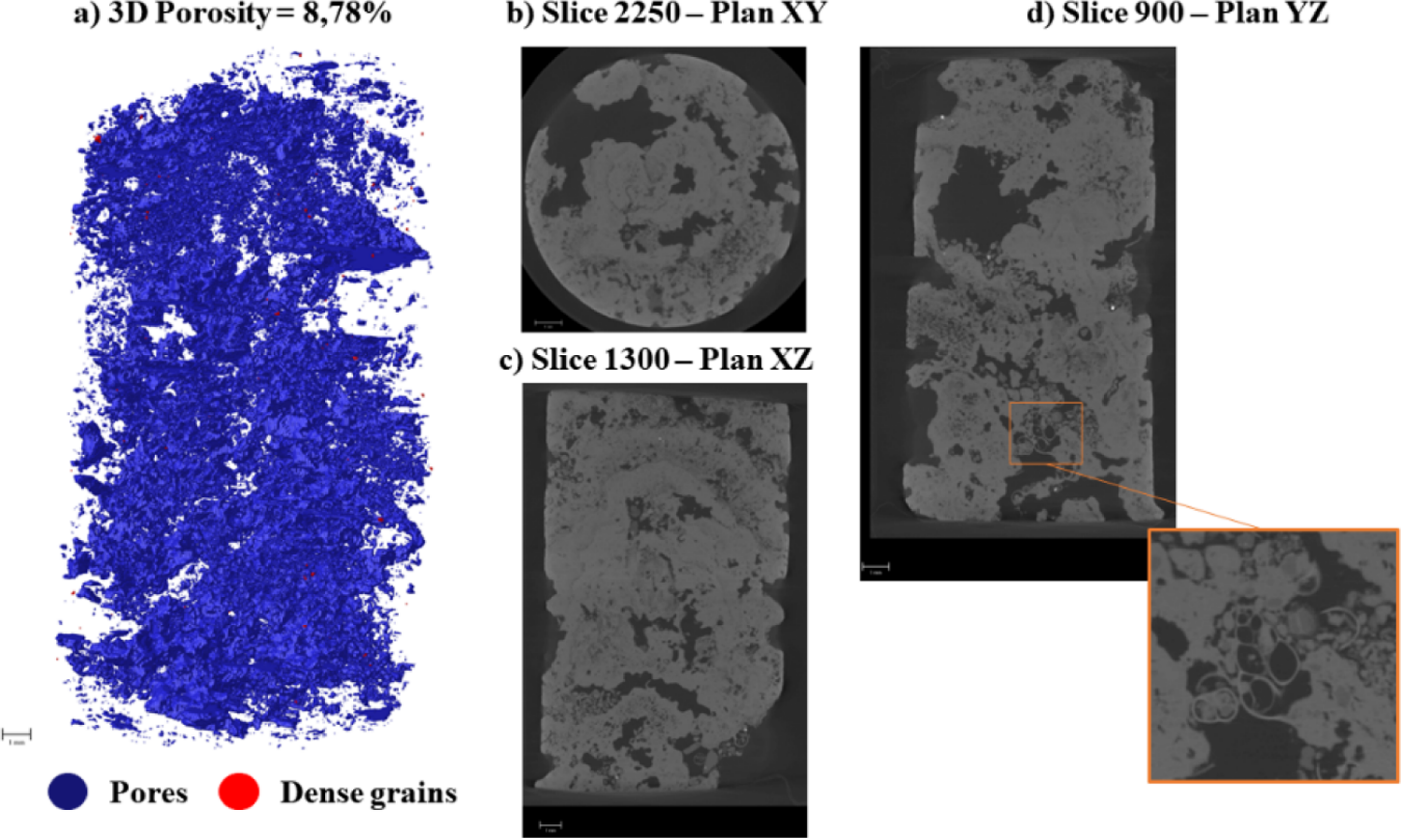
Pore architecture of Facies B resolved by micro-CT multiplanar views.

In Facies C (Figure 6), pores are predominantly elliptical to flattened, widely distributed throughout the sample, and frequently coalesce into vuggy zones. These pore networks are commonly associated with bioturbated intervals, which may act as preferential pathways for fluid flow during reactive processes.

**Figure 6:**
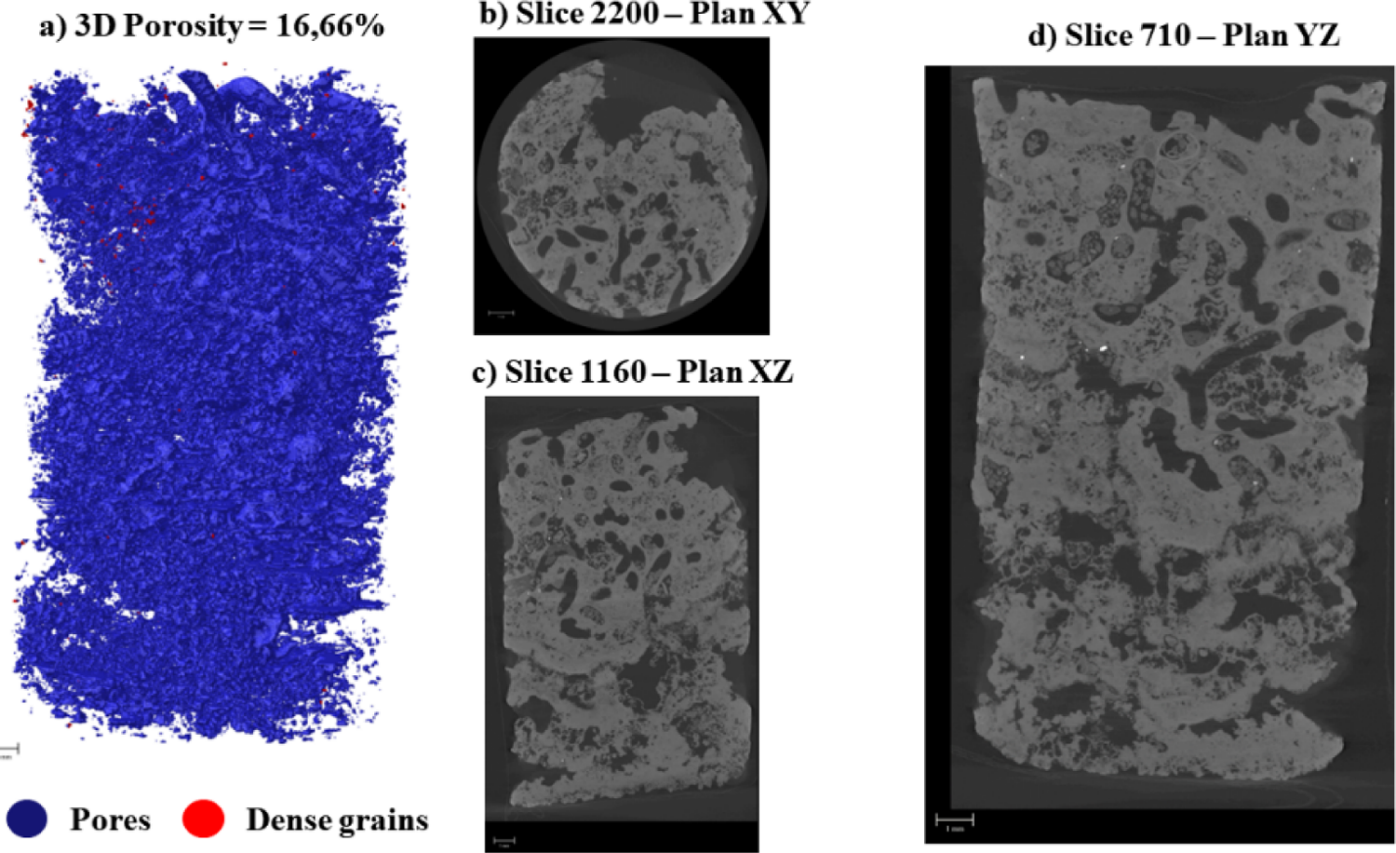
Pore architecture of Facies C resolved by micro-CT multiplanar views.

Figure 7 illustrates the digital porosity, defined as the volumetric fraction of segmented pore space relative to the total scanned sample volume, which includes the carbonate matrix, dense grains, and pores. Porosity was quantified in both three dimensions (total 3D porosity) and two dimensions (slice-by-slice 2D distribution) to capture spatial heterogeneity along the vertical profile of each sample.

**Figure 7:**
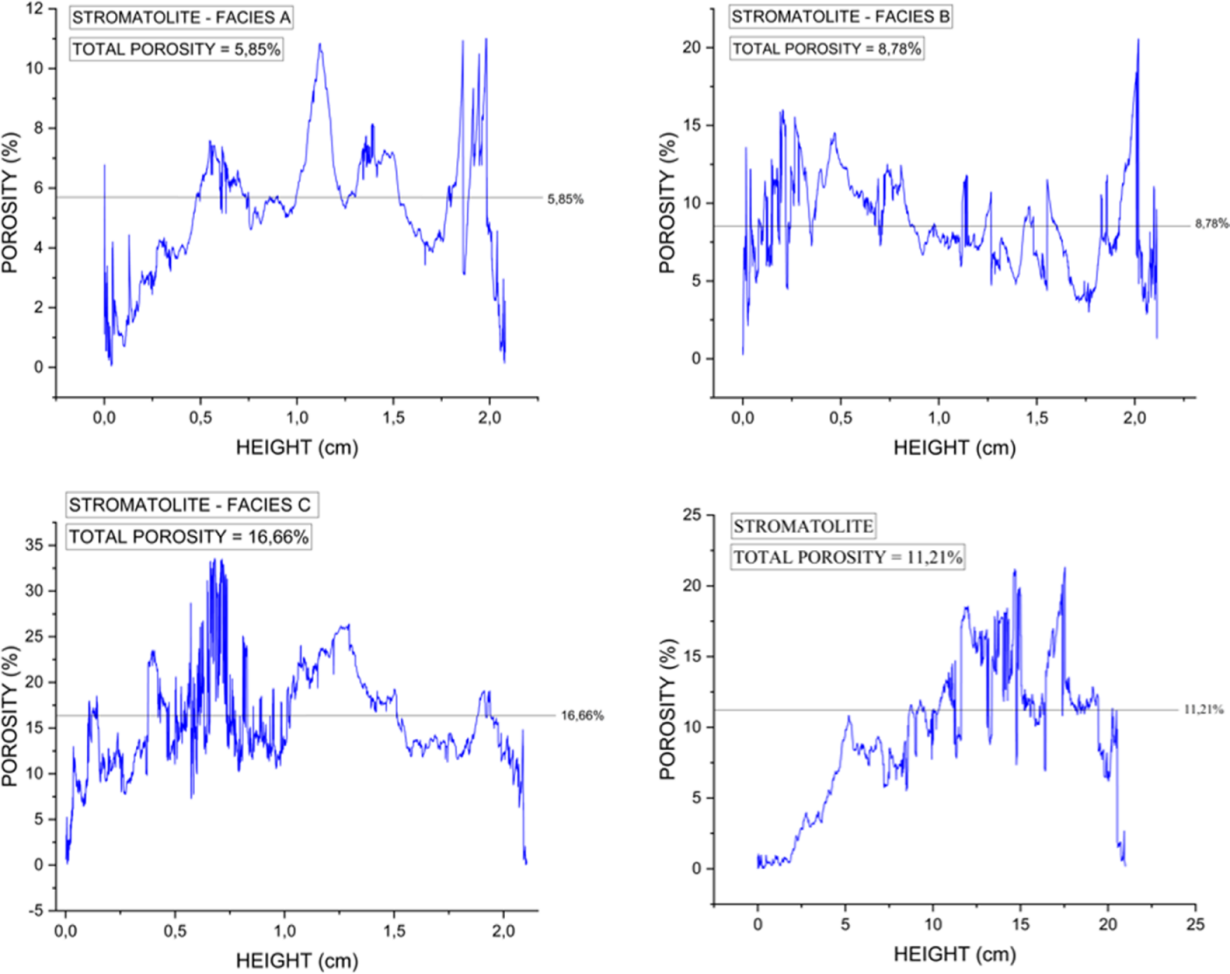
Slice-by-slice 2-D porosity distribution derived from micro-CT segmentation. (a) Facies A; (b) Facies B; (c) Facies C, and; (d) whole-core stromatolite. Horizontal axis = slice number (depth), vertical axis = porosity (%).

The pore distribution profiles indicate that all samples exhibit heterogeneous porosity along their lengths. In Facies A, porosity is relatively low at the base and gradually increases toward the top. In contrast, Facies B and Facies C show more irregular vertical variations, with no consistent trend, reflecting greater internal textural complexity.

The whole-core stromatolite sample displays an overall upward increase in porosity associated with the transition between microbialite facies A, B, and C. In the lower portion of the core, slice-by-slice 2D porosity values are generally lower than the average total 3D porosity, whereas in the middle and upper intervals, local porosity values tend to exceed this average. This behavior highlights the influence of facies-dependent depositional and diagenetic controls, effectively captured by high-resolution digital imaging.

Figure 8 illustrates the comparison between initial digital porosity and magnesian calcite content across the three stromatolitic facies (ST-A, ST-B, and ST-C). The facies display contrasting trends between their structural and mineralogical properties: while ST-C shows the highest digital porosity, ST-A exhibits the lowest. This gradient aligns with previously observed textural differences, where ST-C was characterized by pervasive vuggy porosity, and ST-A exhibited vugs-type pore systems associated preferentially with laminations.

**Figure 8:**
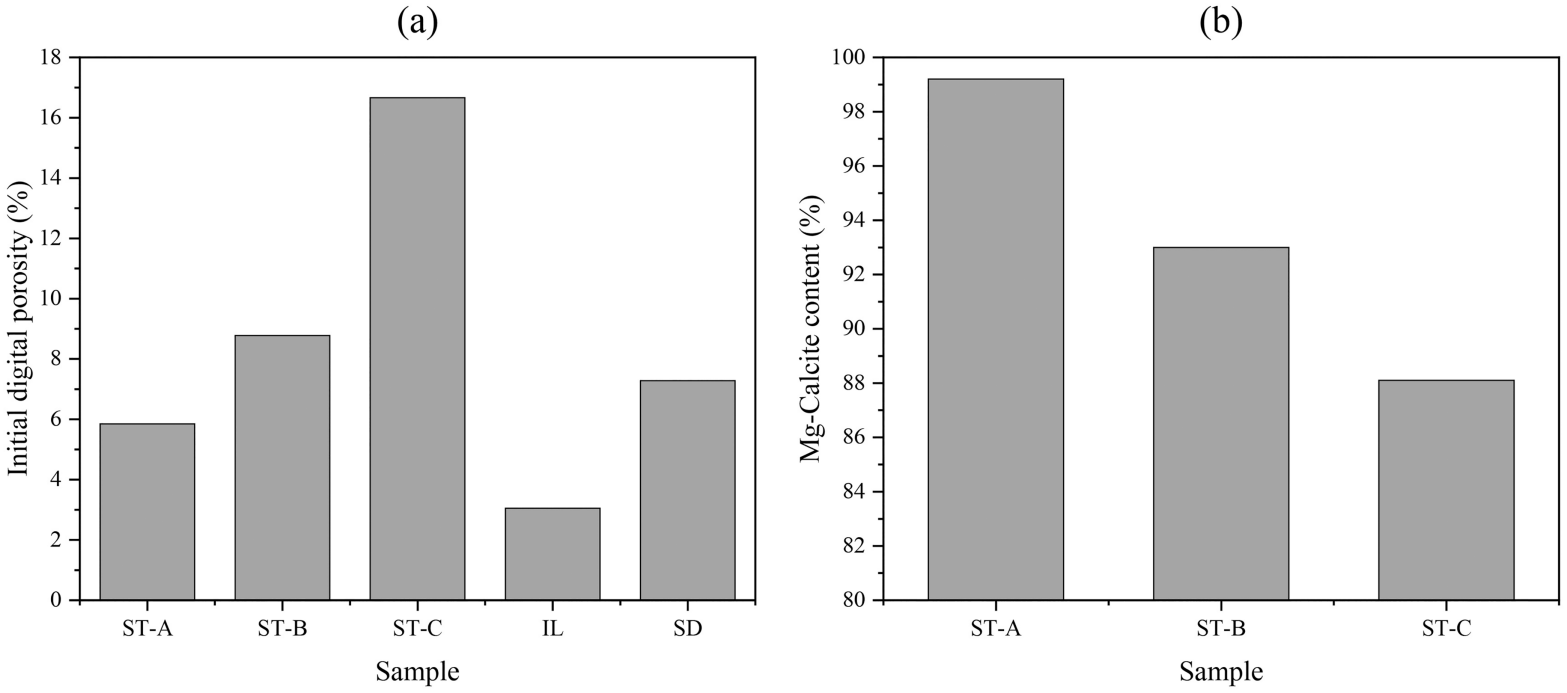
Comparison of initial digital porosity (%) and magnesian calcite content (%) across IL, SD and stromatolitic facies: ST-A, ST-B, and ST-C.

In contrast to the porosity trend, the magnesian calcite content follows an inverse pattern. ST-A presents the highest relative content of Mg-calcite, with a progressive decrease toward ST-C. This mineralogical variation suggests differing degrees of isomorphic substitution within the calcite lattice along the stromatolite column.

The comparison between these two data sets emphasizes a decoupling between structural and compositional characteristics across the stromatolitic intervals. Whereas ST-C is dominated by open and irregular pore networks that enhance fluid accessibility, ST-A is compositionally less reactive but structurally more compact. ST-B, in both parameters, occupies an intermediate position, reflecting its transitional thrombolitic texture and mixed pore morphology.

### 3.2 Static Dissolution Experiments

Figure 9 presents the experimental results of the static acid dissolution tests performed on the carbonate samples using a 0.5 mol·L^–1^ HCl solution. To assess the dissolution behavior of the stromatolitic facies and standard carbonates, mass loss was systematically monitored over time. Panel (a) displays the combined dissolution trends for all samples plotted on a logarithmic time scale, allowing for direct visual comparison of their relative reactivities. Panels (b) through (f) show individual dissolution profiles with corresponding linear regression fits for each rock type, enabling quantification of average dissolution rates and evaluation of kinetic linearity. The values of *t*_50_ and reaction rate obtained for Indiana Limestone and Silurian Dolomite Samples are similar to the values reported in the literature.^28^

**Figure 9:**
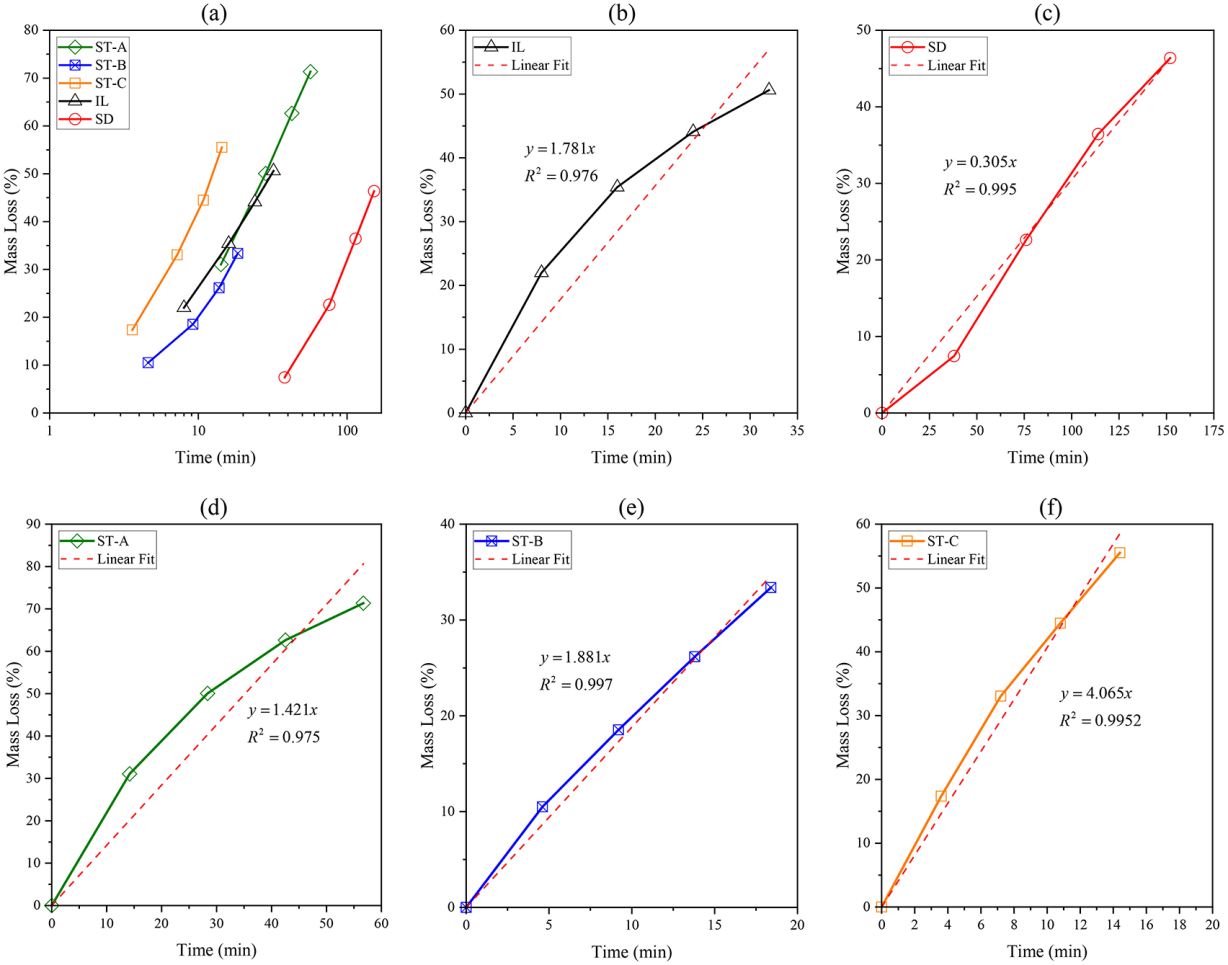
Mass loss (%) over time during static acid dissolution in 0.5 mol·L^–1^ HCl. (a) Combined plot for all samples displayed on a logarithmic time scale. (b–f) Individual linear regression fits for each lithology: (b) Indiana Limestone (IL), (c) Silurian Dolomite (SD), (d) stromatolitic facies ST-A, (e) stromatolitic facies ST-B, and (f) stromatolitic facies ST-C.

The stromatolitic facies exhibit clear differences in dissolution behavior. ST-C displays the highest mass loss over time, followed by ST-B, while ST-A shows the slowest response among the microbialites. Notably, ST-A exhibits a slightly lower dissolution rate compared to Indiana Limestone (IL), which is composed predominantly of pure calcite. In contrast, ST-B and ST-C dissolve at substantially higher rates than IL.

This behavior is nontrivial when considered from a purely mineralogical perspective. Given that Indiana Limestone consists predominantly of calcite and the stromatolitic facies contain magnesian calcite, thermodynamic expectations would predict higher solubility and faster reaction kinetics for IL. The incorporation of Mg^2+^ into the calcite lattice typically reduces its solubility product and enhances structural stability, resulting in slower dissolution compared to pure calcite. Consequently, the faster dissolution observed in ST-B and ST-C relative to Indiana Limestone suggests that dissolution is not governed solely by mineralogical composition. The results suggest that, under the specific conditions evaluated, the dissolution rate is controlled more by microstructural and textural characteristics such as pore architecture and connectivity than by mineralogical composition alone.

As previously demonstrated through micro-CT imaging, ST-B and ST-C possess more open and heterogeneous pore networks than IL. ST-C, in particular, exhibits pervasive vuggy porosity and high internal connectivity, which significantly enhances acid accessibility and promotes continuous exposure of fresh reactive surfaces during the experiment. In contrast, IL, despite its mineralogical favorability, exhibits a more compact interparticle porosity system that may limit effective transport of acid into the sample interior under static conditions.

ST-A, though compositionally enriched in magnesian calcite, also has a relatively compact, laminated internal structure, which restricts acid ingress and leads to slower dissolution, even slower than IL.

Figure 10 complements the time-resolved mass loss data presented earlier (Figure 9) by summarizing two quantitative indicators of reactivity: the average dissolution rate (left panel) and the *t*_50_ value (right panel), defined as the time required for 50% mass loss.

**Figure 10:**
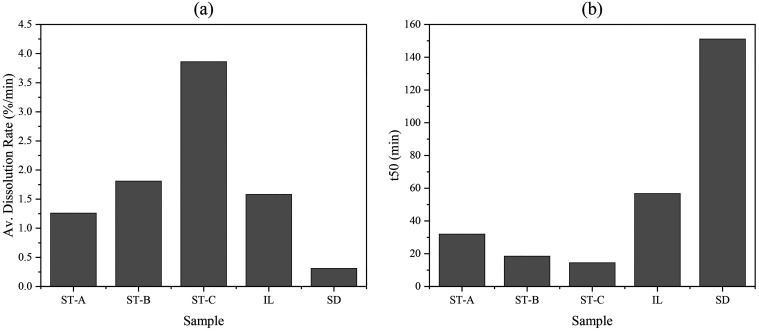
Summary of dissolution kinetics for stromatolitic facies (ST-A, ST-B, ST-C) and standard carbonates (IL = Indiana Limestone, SD = Silurian Dolomite). (a) Average dissolution rate (% mass loss per minute); (b): *t*_50_, defined as the time to 50% mass loss.

A progressive increase in reactivity is observed from Layer A to Layer C. Indiana Limestone exhibits an intermediate dissolution rate, comparable to ST-A, and the Silurian Dolomite displays the lowest reactivity among all samples. These trends are mirrored in the *t*_50_ values, which decrease from Layer A to C, remain moderate for Indiana Limestone, and reach their maximum for the Silurian Dolomite, indicating significantly slower mass loss in the latter.

### 3.3 X-ray Micro-CT Analyses of the Dissolution Experiment

The morphological evolution of the carbonate samples during static acid exposure was assessed through high-resolution microcomputed tomography (micro-CT) imaging across five dissolution cycles. Figures 11 and 12 present three-dimensional volume renderings and corresponding axial slices, respectively, for each sample: stromatolitic facies (Layers A, B, and C), Indiana Limestone (IL), and Silurian Dolomite (SD).

**Figure 11:**
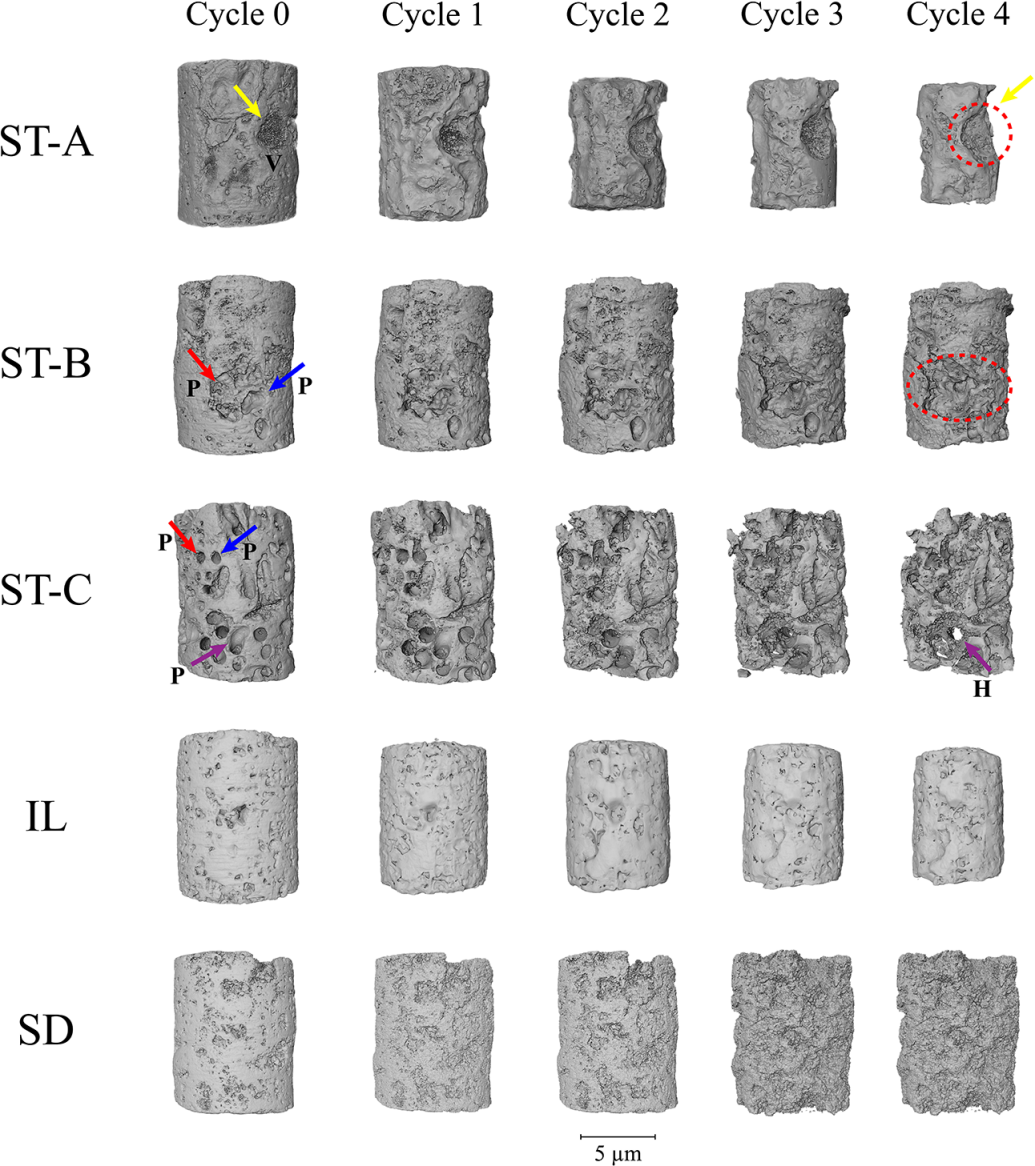
Micro-CT reconstructions of carbonate samples at successive dissolution cycles (Cycle 0 to Cycle 4) under static acid exposure.

**Figure 12:**
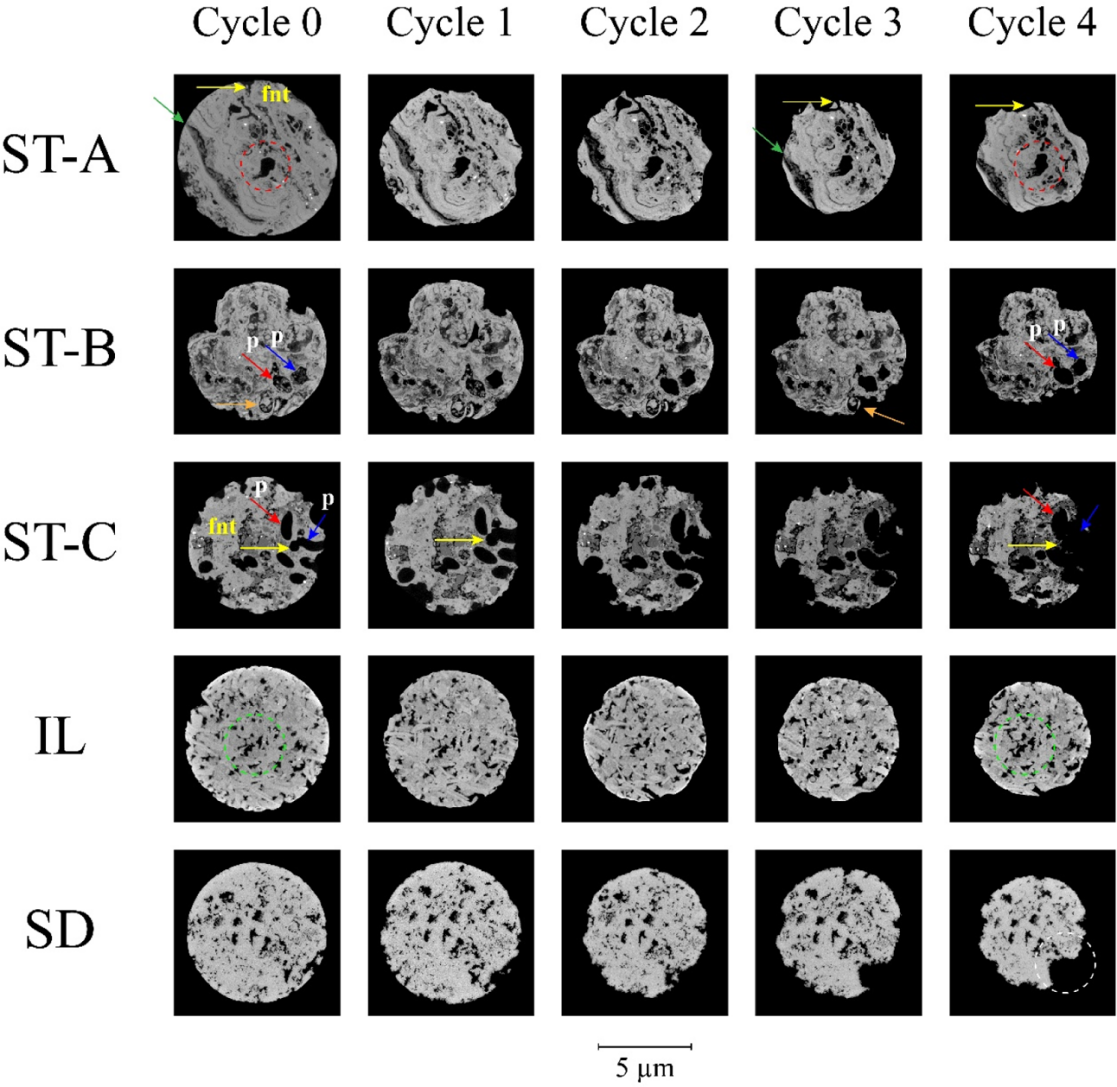
Axial slice images showing the evolution of facies dissolution. ST-A showing the evolution of facial dissolution from fenestral channels (fnt) with their loss of diameter, green arrow pointing to zones of dissolved laminations and red dashed circles showing expansion of vugs. ST-B, highlight for pair of blue and red arrows showing fused pores and orange arrow showing dissolution of microgastropod carapace. ST-C, highlight pair of red and blue arrows showing fused pores (p) from the dissolution of fenestral channels (fnt). Green dashed circles showing interparticle pore dissolution of the Indiana limestone pattern and white dashed circle showing zone with isotropic dissolution at point sites of the Silurian Dolomite pattern.

In Layer A, there is a greater loss of diameter caused mainly by the presence of macrovugs on the surface and microlaminations, affecting the generatrix of the miniplugs. Volume renderings (Figure 11) reveal a small diameter reduction and increased surface-level corrosion near pre-existing vugs (v), while axial sections (Figure 12) show that dissolution occurred mainly in zones with crenulated internal laminations formed by lumpy-textured mycritical to microspastic masses with irregular contours where acid permeability is higher and by small fenestrals (fnt) created by serpulids connected to vugs, which allowed better acid drainage to the innermost areas of the layer reaching the matrix. This subdued response aligns with the low average dissolution rate and longer *t*_50_ previously quantified.

In contrast, Layer B undergoes progressive internal modification. Initially characterized by a thrombolitic texture and isolated mesopores, this sample develops clear signs of pore coalescence (p) and channel interconnectivity across the cycles. Volume images show subtle surface degradation, but axial slices distinctly capture pore enlargement and increased heterogeneity by the final cycle. These microstructural changes are indicative of evolving acid flow pathways and validate the intermediate kinetic response observed in the dissolution experiments.

Layer C exhibits the most pronounced structural evolution. The initial architecture, dominated by vuggy and fenestral porosity, facilitates rapid acid penetration and early stage reactivity. Across successive cycles, the images reveal widespread pore merging, thinning of solid bridges, and eventual collapse of internal laminae and fenestral structures, in addition to the formation of holes in the sample. Both volumetric and axial slices demonstrate highly dynamic reconfiguration of the pore network, confirming that pre-existing macroporosity served as a conduit for aggressive dissolution. The resulting transformation is consistent with the high dissolution rate and the shortest *t*_50_ value among all samples.

Indiana Limestone displays a more restrained structural response. While volume renderings show mild surface dissolution, axial slices confirm that pore size and distribution remain largely unaffected. Slight roughening at the grain contacts is evident in later stages, but the interparticle framework is maintained. These findings support the interpretation that, despite its high calcite content, the limited pore accessibility and homogeneous texture constrain acid penetration under static conditions.

The Silurian Dolomite sample exhibits the lowest degree of structural alteration. Throughout the dissolution cycles, both external and internal morphologies remain virtually unchanged. A slight increase in surface rugosity is detectable in the axial slices by the final cycle, but no internal coalescence or pore development is observed.

## 4 Conclusions

This study provides new insights into the static acid dissolution behavior of stromatolitic carbonates compared with standard carbonate rocks, highlighting the combined influence of mineralogical composition, microstructural architecture, and pore system heterogeneity on reactivity. Static dissolution experiments using 0.5 mol·L^–1^ HCl, integrated with high-resolution microcomputed tomography (micro-CT), allowed a direct evaluation of morphological evolution and dissolution kinetics across stromatolite facies (ST-A, ST-B, ST-C), Indiana Limestone (IL), and Silurian Dolomite (SD).

The main findings of this study can be summarized as follows.

1. Dissolution behavior did not correlate directly with bulk mineralogy. Despite its nearly pure calcite composition, Indiana Limestone exhibited dissolution rates comparable to or lower than stromatolitic facies enriched in magnesian calcite, demonstrating that mineral composition alone does not govern reactivity under static conditions.
2. Microstructural and textural attributes, particularly pore geometry, size distribution, and connectivity, were identified as the dominant controls on dissolution kinetics, as confirmed by micro-CT observations of pore network evolution during acid exposure.
3. Among the stromatolitic facies, reactivity increased systematically from Layer A to Layer C, consistent with increasing macroporosity, internal heterogeneity, and improved pore connectivity. Facies with more open pore networks showed pronounced morphological transformation, whereas laminated and compact facies displayed limited structural alteration.
4. Silurian Dolomite showed the lowest reactivity, characterized by minimal mass loss, negligible pore evolution, and the highest *t*_50_ values, reflecting the combined effect of mineral stability and restricted pore accessibility.

These results emphasize the importance of microstructural heterogeneity when evaluating carbonate dissolution processes in complex reservoir analogs, particularly in Pre-Salt systems.
